# Supplementary material for: Healthcare Resource Use and Costs of Allogeneic Hematopoietic Stem Cell Transplantation Complications: A Scoping Review
Source: Curr Oncol. 2025 May 16;32(5):283. doi: 10.3390/curroncol32050283 (PMC12109855; doi:10.3390/curroncol32050283)
Supplement: Supplementary file 1 [file curroncol-32-00283-s001.zip › curroncol-3497381-supplementary.pdf]

## Supplementary Table S1: MEDLINE search strategy

| Search number | Query |
|---|---|
| #11 | Search: (#9) NOT (#10) Sort by: Publication Date |
| #10 | Search: (animals [mh] NOT humans [mh]) Sort by: Publication Date |
| #9 | Search: (#5) AND (#8) Sort by: Publication Date |
| #8 | Search: (#6) OR (#7) Sort by: Publication Date |
| #7 | Search: Economics[Mesh] Sort by: Publication Date |
| #6 | Search: ((health* resource utili*) OR (economic*)) OR (cost analys*) Sort by: Publication Date |
| #5 | Search: (#3) OR (#4) Sort by: Publication Date |
| #4 | Search: (Bone Marrow Transplantation[Mesh]) OR (Stem Cell Transplantation[Mesh]) Sort by: Publication Date |
| #3 | Search: (#1) AND (#2) Sort by: Publication Date |
| #2 | Search: ((((((((blood and marrow transplant*) OR (stem cell transplant*)) OR (haematopoietic stem cell transplant*)) OR (hematopoietic stem cell transplant*)) OR (haematopoietic cell transplant*)) OR (hematopoietic cell transplant*)) OR (blood transplant*)) OR (haematopoietic progenitor*)) OR (hematopoietic progenitor*)) OR (bone marrow transplant*) Sort by: Publication Date |
| #1 | Search: (allogeneic) OR (allograft) Sort by: Publication Date |

## Supplementary Table S2: Template source of evidence, details, characteristics and results extraction instrument

| Evidence source Details and Characteristics - SUMMARY | | REFERENCE |
|---|---|---|
| Citation details | | |
| Total or partial study extraction | | |
| Type of Study (e.g. single centre, retrospective) | | |
| Country | | |
| Study Objective/Aim | | |
| Analyses plan (e.g., descriptive stats, economic evaluation, budget impact etc) | | |
| Sample Size (total or subpopulation, specify) | | |
| Population Characteristics (including any relevant data capture dates) | | |
| Disease (if not specified above) | | |
| Other | | |
| **Details/Results extracted from source of evidence (in relation to the concept of the scoping review)** | | |
| COMPREHENSIVE APPROACH/SPECIFIC APPROACH - specify: | | |
| Result Timeframe | | |
| Method | | |
| Data source(s) | | |
| Analyses details | | |
| Assumptions | | |
| HRU and/or cost | | |
| Calculations (Units)/Currency | | |
| HRU Units | | |
| Results | | |
| Relevant discussion points | | |
| Limitations | | |

Notes: * Assessment of clinical outcome associated with HSCT: (i) Comprehensive approach or (ii) focused approach (such as GVHD or other allo-HSCT associated complications)

## Supplementary Table S3. Summary of HRU and Cost Study Characteristics

| Author | Allo-HSCT Population | Sample Size | Country | Design | Analysis Summary | Data Source (year) | HRU/Cost | Currency (year) | Timeframe | Extraction | Other |
|---|---|---|---|---|---|---|---|---|---|---|---|
| **Graft versus host disease** | | | | | | | | | | | |
| Lee, 2002 | URD | TCD = 48, IST = 98 | USA | Single Centre | Descriptive analyses | Clinical, trial and administrative databases (Jan 1997 - Dec 1999) | Both | USD (2000) | 1.5 years | entire study cohort | aGVHD prophylaxis - TCD and IST |
| Yerrabothala, 2018 | allo-HSCT with GVHD | 12 | USA | Prospective Single Centre | Time and motion study | Hospital cost-accounting system | Both | dollars (NS) | per procedure | entire study cohort | GVHD - ECP |
| Yu, 2019 | allo-HSCT | aGVHD = 515, non-GVHD = 4,976 | USA | population-based retrospective study | Descriptive analyses | National Inpatient Sample (2009-2013) | Both | dollars (NS) | allo-HSCT admission | partial - subgroup | aGVHD and non-GVHD |
| Yu, 2020 | First allo-HSCT | aGVHD = 906 (SR/HR subgroup = 158), non-GVHD = 1,529 | USA | population-based retrospective study | Descriptive analyses | Premier Healthcare Database (Jan 2011 - June 2016) | Both | USD (NS) | 100 days | entire study cohort | aGVHD (subgroup: HR/SR), non-GVHD |
| Bachier, 2021 | allo-HSCT | 5259 | USA | population-based retrospective study | Descriptive analyses | Medicare 5% claims database (2013-2016) & PharMetrics database (2013-2018) | Both [(a)] | dollars (2016) | 12 months post cGVHD diagnosis | entire study cohort | cGVHD |
| Boluda, 2021 | allo-HSCT with steroid-refractory of steroid-dependent cGVHD | ECP = 20, non-ECP = 20 | Spain | Retrospective Single Centre (matched cohort) | Descriptive analyses | NS (Jan 2010-May 2016) | Both | Euro (2016) | 1 year (or refractory) from steroid-refractory cGVHD | entire study cohort | cGVHD - ECP vs non-ECP |
| Farhadfar, 2021 | First allo-HSCT | aGVHD = 174 early onset, 11 late onset; non-aGVHD = 106 | USA | Retrospective Single Centre | Descriptive analyses | Health records and administrative data (aGVHD: Jul 2010 - Jan 2019, non-aGVHD: Jul 2010 - Jan 2018) | Both | USD (2018) | early phase: 100 days; late phase Day 101-365 | entire study cohort | aGVHD |
| Schain, 2021 | allo-HSCT who survived ≥ 182 days | Mild cGVHD = 345, moderate-severe cGVHD = | Sweden | population-based | Descriptive analyses | National Registers (2006-2017) | Both [(b)] | Euro (2018) | 10 year observation time (index date = 182 days post HSCT – | entire study cohort | cGVHD |

| Author | Allo-HSCT Population | Sample Size | Country | Design | Analysis Summary | Data Source (year) | HRU/Cost | Currency (year) | Timeframe | Extraction | Other |
|---|---|---|---|---|---|---|---|---|---|---|---|
| | | 551, non-cGVHD = 350 | | retrospective study | | | | | also reported outcomes for first 3 years of follow-up) | | |
| Bell, 2022 | GVHD diagnosis following allo-HSCT | 689 | USA | population-based retrospective study | Descriptive analyses | Optum Research Database (Jul 2010 - Aug 2019) | Both | dollars (2019) | 2 years (index date = date of first claim for systemic steroids post GVHD diagnosis) | entire study cohort | GVHD (steroid-related complications) |
| Sabatelli, 2022 | First allo-HSCT with subsequent aGVHD | 55 | Finland and Sweden | Retrospective Multi Centre | Descriptive analyses | Medical records (Jan 2016 - Jun 2017) | HRU | • | Data period | entire study cohort | moderate and severe aGVHD |
| Scheid, 2022 | allo-HSCT | cGVHD = 165, without GVHD = 35 | Germany | population-based retrospective study | Descriptive analyses | German Claims Database (2013-2018) | HRU | • | 3 years (index date = first day of HSCT hospitalization) | entire study cohort | cGVHD |
| **Cytomegalovirus** | | | | | | | | | | | |
| Robin, 2017 | First allo-HSCT | 208 | France | Retrospective Single Centre | Descriptive analyses | Hospital billing system (Jan 2006 - Dec 2013) | Both | Euro (2015) | 12 months | entire study cohort | Pre-ET strategy: CMVi or CMVd |
| CADTH, 2018 | CMV-seropositive allo-HSCT | NA | Canada | Pharmacoeconomic Review Report | | Canadian cost databases and literature | Cost | NS | Lifetime | entire study cohort | CMV prophylaxis - letermovir vs usual care [(c)] |
| Hakimi, 2018 | allo-HSCT with CMVd | CMVd (exposed) = 165, without CMVd (non-exposed) = 330 | France | Retrospective matched cohort study | Descriptive analyses | Programme de Me´ dicalisation des Syste` mes d'Information hospitalization database in France | Both | Euro (NS) | 12 months (index date: exposed = CMVd diagnosis, non-exposed = allo-HSCT admission) | entire study cohort | With and without CMVd matched 1:2 |
| Webb, 2018 | allo-HSCT | 300 | USA | Retrospective Single Centre | Descriptive analyses | Medical Records (Jan 2006 - Aug 2015) | Cost | USD (NS) | 100 days | partial - study arm | Pre-ET for CMVi, and CMVd - CMV seropositive recipients received IV acyclovir |
| Huang, 2019 | First allo-HSCT | CONV = 88, T-cell depleted = 230 | USA | Single Centre | Descriptive analyses | Medical records and hospital research | HRU | • | 180 days | entire study cohort | Pre-ET - comparison of conventional and CD-34 selected HSCT with varying CMV status. Participants received |

| Author | Allo-HSCT Population | Sample Size | Country | Design | Analysis Summary | Data Source (year) | HRU/Cost | Currency (year) | Timeframe | Extraction | Other |
|---|---|---|---|---|---|---|---|---|---|---|---|
| | | | | | | databases (Jun 2010 - Dec 2014) | | | | | acyclovir for HSV and VZV prophylaxis. |
| NICE, 2019 | CMV-seropositive allo-HSCT | NA | UK | Health Technology Assessment | | NHS Reference Costs and literature | Cost | Pound (NS) | Lifetime | entire study cohort | CMV prophylaxis - letermovir vs standard of care (placebo) |
| Restelli, 2019 | CMV-seropositive allo-HSCT | NA | Italy | Economic Evaluation | | Clinical trials data and literature | Cost | Euro (2018) | Lifetime | entire study cohort | CMV prophylaxis - letermovir vs no prophylaxis |
| Ueno, 2019 | First allo-HSCT | No CMV episode = 177, CMV episode = 739 | Japan | Retrospective cohort study | Descriptive analyses | Claims Data (Apr 2010 - Mar 2018) | Both | USD (2018) | 180 days | entire study cohort | CMV Management |
| Chan, 2020 | CMV-seropositive allo-HSCT | NA | Hong Kong | Economic Evaluation: decision analytical model | | Clinical trials data, single-center cost data | Cost | HKD (NS) | Lifetime | entire study cohort | CMV prophylaxis - letermovir vs Pre-ET |
| Chen, 2020 | allo-HSCT | 12 | USA | Retrospective Single Centre | Descriptive analyses | Medical records (Apr 2014 - Dec 2015) | Both | dollars (2017) | NS | partial - study arm | Pre-ET with foscarnet |
| El Haddad, 2020 | allo-HSCT (i) CMV-seropositive admitted for/with CMVi (ii) admission for/with GVHD | CMV = 100, control = 50 | USA | Retrospective cohort study | Descriptive analyses | Vizient Database, medical records and Department database (Jan 2012 - Dec 2015) | Both | dollars (NS) | 1 year | entire study cohort | Pre-ET for CMVi vs GVHD admissions (without CMVi). Prophylaxis for CMV included ganciclovir, foscarnet and valacyclovir |
| Fang, 2020 | CMV-seropositive - first allo-HSCT | pre-emptive = 208, no pre-emptive = 149 | USA | Retrospective Single Centre | Descriptive analyses | Medical record and hospital database linked through Vizient Database | Both | USD (2017) | 180 days | entire study cohort | Pre-ET. Acyclovir prophylaxis for HSV and VZV |
| Peffault De Latour, 2020 | CMV-seropositive allo-HSCT | 572 | France | Retrospective Multi Centre | Descriptive analyses | Health records (Jan 2010 - Dec 2014) | Both | Euro (NS) | 1 year | entire study cohort | CMVi |
| Saullo, 2020 | First allo-HSCT | csCMVi = 170, without csCMVi = 218 | USA | Retrospective Single Centre | Descriptive analyses | Clinical database, medical records (2009 - 2013) | Both | USD (2018) | 1 year | entire study cohort | Pre-ET - patients -/+ clinically significant CMVi. Herpes virus prophylaxis with acyclovir |

| Author | Allo-HSCT Population | Sample Size | Country | Design | Analysis Summary | Data Source (year) | HRU/Cost | Currency (year) | Timeframe | Extraction | Other |
|---|---|---|---|---|---|---|---|---|---|---|---|
| Schelfhout, 2020a | First allo-HSCT | no CMV readmission = 1610, 1 CMV readmission = 161, 2+ CMV readmission = 51 | USA | Retrospective cohort study | Descriptive analyses | Premier Healthcare Database (Jan 2006 - Mar 2015) | Both | dollars (NS) | 100 days | entire study cohort | CMVi- no vs 1 vs 2+ CMV readmission. |
| Schelfhout, 2020b | First allo-HSCT | with CMV = 410, without CMV = 1415 | USA | Retrospective cohort study | Descriptive analyses | IBM MarketScan Database (Jan 2010 - Apr 2015) | Both | USD (2016) | 1 year | entire study cohort | CMVi |
| Alsumali, 54869 | CMV-seropositive allo-HSCT | NA | USA | Economic Evaluation: Decision model | | Trials Data and literature | Cost | USD (2019) | Lifetime | entire study cohort | CMV prophylaxis - letermovir vs no prophylaxis. Assumes clinically significant CMVi received Pre-ET |
| Golan, 2021 | CMV-seropositive allo-HSCT | letermovir = 325, placebo = 170 | NS | Multicenter | Descriptive analyses | phase III, multicenter, double-blind, placebo-controlled, randomized trial | HRU | • | 48 weeks | entire study cohort | CMV prophylaxis - impact of letermovir on re-hospitalization |
| Ranti, 2022 | First allo-HSCT | No CMV infection = 103, CMV infection = 148 | Finland | Retrospective Single Centre | Descriptive analyses | Hospital data (Jan 2013 - Dec 2018) | HRU | • | 1 year of follow-up | entire study cohort | burden of disease associated with clinically significant CMV |
| **Fungal Infections** | | | | | | | | | | | |
| Sánchez-Ortega, 2013 | first allo-HSCT | posaconazole = 33, itraconazole = 16 | Spain | Single Centre | Descriptive analyses | Hospital records (Aug 2005 - Mar 2009) | Cost | Euro (2008) | 100 days | entire study cohort | prophylaxis - posaconazole (prospective review) vs itraconazole (retrospective review) |
| Bertz, 2016 | allo-HSCT | 106 | Germany | Prospective Single Centre | Descriptive analyses | Jan - Dec 2010 | Both | Euro (2010) | NS, discharge or transfer to ICU | entire study cohort | prophylaxis |
| Walker, 2019 | allo-HSCT | NA | USA | Economic Evaluation - state transition model | | Literature | Cost | USD (2019) | 100 days | entire study cohort | prophylaxis, pre-emptive or empirical |
| Ueno, 2021 | allo-HSCT | 863 | Japan | population-based retrospective study | Descriptive analyses | Hospital-based claims database (Jan 2010 - Jan 2019) | Both | USD (2019) | 360 days (index date = hospital admission for allo-HSCT) | partial - study arm | Stratified by 4 different antifungal regimens and no antifungal |

| Author | Allo-HSCT Population | Sample Size | Country | Design | Analysis Summary | Data Source (year) | HRU/Cost | Currency (year) | Timeframe | Extraction | Other |
|---|---|---|---|---|---|---|---|---|---|---|---|
| Wingen-Heimann, 2021 | allo-HSCT | Posaconazole + micafungin = 97, micafungin = 216 | Germany | Pharmacoeconomic Evaluation (single-center cohort analysis) | | Cologne Cohort of Neutropenic Patients Register and Hospital information system (Jan 2010 - Dec 2015) | Both | Euro (2016) | initial inpatient stay with allo-HSCT | entire study cohort | prophylaxis - posaconazole + micafungin vs micafungin |
| **Virus associated Hemorrhagic Cystitis** | | | | | | | | | | | |
| Gilis, 54989 | allo-HSCT | with BKV-HC = 43 | France | Single Centre | Descriptive analyses | NS (Jan 2007 - Dec 2011) | Both | Euro/dollars (NS) [d] | 5 years | partial - study arm | Burden of BKV-associated HC |
| McGuirk, 2021 | allo-HSCT | with V-HC = 606, without V-HC = 10,900 | USA | Retrospective cohort study | Descriptive analyses | US Claims Data (Jan 2012 - Dec 2017) | Both | USD (2019) | 1 year | partial - subgroup | V-HC vs no V-HC |
| **Acute Respiratory Tract Infection** | | | | | | | | | | | |
| Wattanakamolkul, 2022 | allo-HSCT | allo-HSCT ARTI = 49, allo-HSCT non-ARTI = 65, Cord ARTI = 37, Cord non-ARTI = 34 | Japan | Retrospective cohort study | Descriptive analyses | JMDC Claims Data (Jul 2017 - Dec 2018) | Both | JPY (2017) | post-HSCT hospitalization: HSCT (index month) to discharge month | partial - subgroup | ARTI and non-ARTI during post-HSCT hospitalization |

Notes: (a) Cost measures did not meet criteria for data analyses in this paper (i.e. average annual cost, total non-pharmacy cost), (b) HRU did not meet criteria for data analyses in this paper (i.e. time in healthcare (inpatient and outpatient), days, (c) Initiation of Pre-ET when appropriate, or treatment of CMVd (d) costs expressed in euros and dollars, however dollar currency not specified, therefore euro costs were used in this analysis

Abbreviations: aGVHD = acute graft versus host disease; allo-HSCT = allogeneic hematopoietic stem cell transplant; ARTI = acute respiratory tract infection; BKV-HC = BK virus associated hemorrhagic cystitis; CADTH = Canadian Agency for Drugs and Technologies in Health; cGVHD = chronic graft versus host disease; CMV = Cytomegalovirus; CMVd = Cytomegalovirus disease; CMVi = Cytomegalovirus infection; CONV = conventional hematopoietic stem cell transplant; ECP = Extracorporeal Photopheresis; GVHD = graft versus host disease; HKD = Hong Kong dollars; HRU = health resource utilization; HSV = Herpes simplex virus; ICU = intensive care unit; IST = immunosuppressive medications; JPY = Japanese yen; NICE = National Institute for Health and Care Excellence, United Kingdom; NA = not applicable; NHS = National Health Service; NS = not specified; Pre-ET = pre-emptive therapy; SR/HR = steroid-refractory or high-risk; TCD = t-cell depletion; UK = United Kingdom; URD = unrelated donor, USA = United States of America; USD = United States dollars; V-HC = virus associated hemorrhagic cystitis; VZV = Varicella zoster virus

## Supplementary Table S4. Summary of HRU and Cost measures reported in the included studies

| Studies | Timeframe | HRU | Costs |
|---|---|---|---|
| **Graft versus host disease** | | | |
| **GVHD Prophylaxis – (TCD cf. IST)** | | | |
| Lee, 2002 | 1 year | - ↓ length of initial hospitalization (26 and 34 days)<br>- ↓ overall hospitalization (39 and 46 days) | - ↓ initial cost ($139,619 and $183,452)<br>- ↓ overall hospitalization cost ($225,663 and $251,638) |
| **aGVHD (cf. non-GVHD)** | | | |
| | | | |
| Yu, 2019; Yu, 2020; Sabatelli, 2022 | Initial hospitalization | • initial hospitalization<br>- ↑ aGVHD (31-38 compared to 24-25 days; SR/HR aGVHD 46 days)<br>- ↓ for one line compared to ≥2 lines (22.5 and 56.5 days; overall aGVHD population 41.3 days) | • initial hospitalization cost<br>- ↑ aGVHD) $169,270- $174,817 compared to $103,599-$107,181; SR/HR aGVHD $226,516) |
| Farhadfar, 2021; Yu, 2020 | 100 days | • % and length of re-admission<br>- ↑ aGVHD (59.2-78.3% and 7 days, compared to 28.3-37.9% and 5 days; SR/HR aGVHD 8 days, respectively)<br>• ICU admission<br>- ↑ aGVHD (13.2-40.6% compared to 6.0-25.4%)<br>• LOS<br>- ↑ aGVHD (35.2 compared to 25.2 days) | • total cost<br>- ↑ aGVHD ($259,066 compared to $189,397)<br>• readmission cost<br>- ↑ aGVHD ($20,187 compared to $14,700; SR/HR aGVHD $23,590)<br>• aGVHD-attributed costs<br>- ↑ aGVHD ($69,669 compared to $20,043)<br>• hospitalization cost<br>- ↑ aGVHD ($232,215 compared to $163,664)<br>• outpatient cost<br>- ↑ aGVHD ($26,851 compared to $25,733) |
| Sabatelli, 2022 | Data period | • % of readmission<br>- ↑ for one line compared to ≥2 lines (91.7% and 87.1% overall aGVHD population 89.1%)<br>• number of hospitalizations<br>- ↓ for one line compared to ≥2 lines (2.3 and 3.4 days overall aGVHD population 2.9 days)<br>• % ICU admission and days<br>- ↓ for one line compared to ≥2 lines (4.5% and 0.6 days compared to 22.2% and 1.6 days, respectively; overall aGVHD population 14.3% and 1.1 days)<br>• % outpatient or ED visit<br>- ↓ for one line compared to ≥2 lines (95.5% and 87.1%; overall aGVHD population 90.9%)<br>• number of outpatient visits per year<br>↓ one line compared to ≥2 lines (12 and 11.4; overall aGVHD population 11.7 days) | |
| **cGVHD (cf. non-GVHD)** | | | |
| Bachier, 2021 | 12 months post cGVHD diagnosis | • % and length of systemic therapy<br>- ↑ for one line compared to ≥4 lines (95.7% and 161 days compared to 29.2% and 106 days, respectively)<br>• number of outpatient visits for cGVHD<br>- 18 visits | |
| Scheid, 2022; Schain, 2021 | 3 years | • % additional cGVHD therapies combined with systemic<br>- ranged from 12-3% for 1-6 additional therapy; highest proportion (36%) required 2 additional therapies<br>• % and number of hospitalizations<br>- ↑ cGVHD (90% and 4.7 compared to 44% and 3.8, respectively) | • total cost<br>- ↑ cGVHD severity (cGVHD moderate/severe – $88,592, mild – $58,141, non-GVHD – $40,286) |

| | | | |
|---|---|---|---|
| | | - ↑ cGVHD severity (cGVHD severe – 97% and 6.2, moderate – 92% and 4.2, and mild – 89% and 3.3, respectively)<br>• LOS for cGVHD<br>- ↑ cGVHD (15 compared to 12 days)<br>- ↑ cGVHD severity (cGVHD severe – 22.1, moderate – 19.5, and mild – 13.1 days) | |
| **GVHD treatment** | | | |
| Yerrabothala, 2018; Boluda, 2021 | Procedure time and 1 year post SR cGVHD | ECP (cf non-ECP)<br>• % and number of hospitalizations<br>- ↓ ECP (44% and 1.4 compared to 58% and 1.7, respectively)<br>• LOS<br>- ↓ ECP (4 compared to 20.5 days)<br>• Outpatient visits and external consult<br>- ↑ ECP (3 and 15 compared to 1 and 7 visits, respectively)<br>• Procedure time<br>- 170 and 340 minutes based on the integrated or multistep ECP systems, 117 and 161 minutes for the CELLEX and UVAR XTS systems | ECP (cf non-ECP)<br>• inpatient cost<br>- ↓ ECP ($22,761 compared to $32,731)<br>• total cost<br>- ↓ ECP ($30,426 compared to $36,215)<br>• cost of ECP procedure<br>- $3,912 |
| Bell, 2022 | 2 years | Systemic steroids for GVHD<br>• % hospitalization<br>- 66% (at least once)<br>• LOS<br>- 20 days (median) | Systemic steroids for GVHD<br>• Hospitalization cost<br>- %157,364<br>- ↑ presence of both acute and chronic GVHD ($208,203 compared to aGVHD or cGVHD alone $205,822 and $126,662, respectively) |
| **Cytomegalovirus** | | | |
| **CMV Prophylaxis – (letermovir cf. SOC or placebo)** | | | |
| Golan, 2021 | 48 days | • % and days of re-admission<br>- ↓ letermovir (55.7% and 18 days, compared to 60.6% and 20.7 days, respectively) | |
| CADTH, 2018; NICE, 2019; Restelli, 2019; Chan, 2020; Alsumali, 2021 | Lifetime for most studies | | • total cost<br>- ↑ letermovir ($19,400-$52,793 compared to $7,948-$42,354)<br>• CMV-related readmission cost<br>- ↓ letermovir ($210-$1,453 compared to $581-$3,838)<br>• CMV prophylaxis cost<br>- ↑ letermovir ($16,222-$16,631 compared to $0)<br>• CMV Pre-ET cost<br>- ↓ letermovir ($683-$2,183 compared to $1,684-$5,381)<br>• CMV disease cost<br>- ↓ letermovir ($72-$228 compared to $84-$267) |
| **CMV infection or disease – (CMV cf comparator)** | | | |
| Fang, 2020; Peffault De Latour, 2020; Schelfhout, 2020 | Initial hospitalization | • initial hospitalization<br>- ↑ CMV group (35.4 pre-ET compared to 31 days no pre-ET; 56.7 CMVi and 36.1 days without-CMVi)<br>- ↑ for one compared to ≥2 CMV-associated readmissions (30.7 and 29.2 days, respectively; 27.1 days for no readmission)<br>• length of ICU admission<br>- ↓ for one compared to ≥2 CMVi (17.6 compared to 21.6 days, respectively; 14.9 days for no CMV) | • initial hospitalization cost<br>- ↑ CMV group ($221,124 Pre-ET compared to $177,057 no Pre-ET) |

| | | | |
|---|---|---|---|
| Webb, 2018; Schelfhout, 2020; Saullo, 2020 | 100 days | • LOS<br>- ↓ for one compared to ≥2 CMV-associated readmissions (31.9 and 52.4 days, respectively; 13.0 days for no readmission) | • total cost<br>- ↑ CMV group ($150,007 CMVi/CMVd compared to $127,576 no CMV)<br>- ↓ for one compared to ≥2 CMVi/CMVd ($122,928 compared to $202,466, respectively; $50,681 no CMV)<br>• antiviral therapy cost<br>- ↑ CMV group ($14.87 for CMVi and $21.73 for CMVd compared to $14.69 for no CMV)<br><u>CMVi within 100 days (cf post-100 days)</u><br>• total cost<br>- ↓ CMVi within 100 days ($146,938 compared to $161,520) |
| Huang, 2019; Fang 2020; Ueno, 2019 | 180 ys | • LOS<br>- ↑ CMV group (93.6 CMVi/CMVd compared to 55.9 days no CMV; 49.8 CMVi compared to 38.1 days no pre-ET)<br>• % and length of readmission<br>- ↑ CMV group (54.8% and 26.4 pre-ET compared to 34.2% and 20.8 days no pre-ET)<br>• % outpatient visits<br>- ↓ for one compared to ≥2 CMVi or CMVd (69.1% compared to 76.6%, respectively; 54.2% for no CMV)<br><u>Conventional HSCT (cf CD34-selected HSCT)</u><br>• LOS<br>- CMV seronegative: ↓ conventional HSCT (23 compared to 21 days)<br>- CMV seropositive with and without viremia: ↑conventional HSCT (32.5 and 31 days compared to 27 and 25 days, respectively)<br>• length of readmissions<br>- CMV seropositive with viremia: ↓conventional HSCT (5 and 8 days, both groups had one readmission)<br>- CMV seronegative and CMV-seropositive without viremia: no readmissions<br>• duration of antiviral therapy<br>- Valganciclovir: ↓ conventional HSCT (34.7 and 56.5 days)<br>Foscarnet: ↑ conventional HSCT (30.2 and 29.3 days) | • total cost<br>- ↓ for one compared to ≥2 CMVi/CMVd ($131,711 compared to $147,614, respectively; $139,888 for CMV population and $86,160 no CMV)<br>• hospitalization cost<br>- ↑ CMV group ($347,425 for CMVi compared to $240,303 no CMV)<br>• readmission cost<br>- ↑ CMV group ($230,442 for Pre-ET compared to $184,776 no Pre-ET) |
| Robin, 2017; Hakimi, 2018; Ueno, 2019; Saullo, 2020; El Haddad, 2020; Schelfhout, 2020; Ranti, 2022; Peffault De Latour, 2020 | 1 year | • LOS<br>- ↑ CMV group (23.1-113.9 CMVi/CMVd compared to 5-87.5 days no CMV[a]; 70.1 days for CMVd)<br>- ↓ for one compared to ≥2 CMVi (27 and 75 days, respectively; 14 days for no CMV)<br>• % and number of readmissions<br>- ↑ CMV group (28.0-95.3% and 1-3.3 CMVi/CMVd compared to 14.7-89.9%, and 0-2.3 no CMV, respectively; 4 CMVd compared to 3.4 no CMV)<br>- ↓ for one compared to ≥2 CMVi (88.2% and 3 compared to 100% and 6, respectively; 89.8% and 3 for CMVi population; 72.8% and 2 for no CMV)<br>• duration of readmission<br>- ↑ CMV group (51.1 and 49.4 for CMVi during and after allo-HSCT hospitalization, compared to 27.2 days without CMVi)<br>• % and length of ICU admission<br>- ↑ CMV group (28.2% compared to 21.6% no CMV)<br>- ↑ for one compared to ≥2 CMVi (12.6% compared to 4.8%; 6.8% for no CMV)<br>• % and number outpatient visits | • total cost<br>- ↑ CMV group ($128,701-$804,956 CMVi/CMVd compared to $13,748-$549,793 no CMV)<br>- ↓ for one compared to ≥2 CMVi/CMVd ($141,277 compared to $168,591, respectively; $134,508 no CMV)<br>• hospitalization cost<br>- ↑ CMV group ($82,857 for CMVi compared to $75,892 no CMV; $604,339 for CMVi/CMVd compared to $411,450 no CMV; $71,279 for CMVd compared to $62,697 no CMV)<br>• Outpatient cost<br>- ↑ CMV group ($168,061 for CMVi/CMVd compared to $112,098 no CMV)<br>-<br><u>CMV seronegative (cf seropositive) recipients</u><br>• total cost<br>- ↑ CMV seronegative ($141,376 compared to $139,496)<br>-<br><u>CMVi during allo-HSCT hospitalization (cf CMVi post-allo-HSCT hospitalization)</u><br>• readmission cost |

| | | | |
|---|---|---|---|
| | | - ↑ CMV group (93.1% and 34.5 CMVi/CMVd compared to 96.8% and 42.2 without CMVi/CMVd, respectively)<br>• % antiviral therapy (Pre-ET)<br>- ↓ for one compared to ≥2 CMVi (98.0% compared to 100%)<br>• % and number of doses of other CMV directed therapy<br>- Intravenous immunoglobulin: 11.2% and 3.4 doses (mean)<br>CMV immunoglobulin: 5.3% and 2.3 doses (mean) | - ↓ CMVi during allo-HSCT hospitalization ($34,315 compared to $37,055; $19,282 for no CMVi) |
| Chen, 2020 | NS | • drug therapy<br>- Foscarnet: 28 days | • hospitalization cost<br>- $29,464 for CMVi |
| **Fungal Infection** | | | |
| **Fungal Prophylaxis** | | | |
| Wingen-Heimann, 2021 | Initial hospitalization | Posaconazole + micafungin (cf. micafungin alone)<br>• initial hospitalization<br>- ↑ posaconazole + micafungin (42 compared to 40 days)<br>• duration of antifungal prophylactic therapy<br>- ↓ posaconazole + micafungin (19 of posaconazole and 15 days of micafungin compared to 21 days of micafungin alone) | Posaconazole + micafungin (cf. micafungin alone)<br>• initial hospitalization cost<br>- ↑ posaconazole + micafungin ($32,049 compared to $31,784)<br>• antifungal prophylaxis drug cost<br>- ↑ posaconazole + micafungin ($8,641 compared to $8,629)<br>• cost of diagnostic approaches<br>- ↑ posaconazole + micafungin ($1,316 compared to $1,205) |
| Sánchez-Ortega, 2013; Walker 2019 | 100 days | | Posaconazole (cf itraconazole)<br>• total cost<br>- ↑ posaconazole ($93,627 compared to $90,647)<br>• antifungal prophylaxis drug cost<br>- ↑ posaconazole ($18,853 compared to $9,941)<br>• inpatient cost<br>- ↓ posaconazole ($62,882 compared to $65,733)<br>-<br>Different antifungal management strategies<br>• total cost<br>- Posaconazole prophylaxis: $170,347<br>- empirical treatment with voriconazole following fluconazole prophylaxis: $165,023<br>- Pre-ET with voriconazole following fluconazole prophylaxis based on regular galactomannan testing: $165,723 and $165,502 based on optical density index of ≥ 0.5 and 1.0, respectively |
| Bertz, 2016 | NS, discharge or transfer to ICU | | • total cost of diagnostics<br>- $709 for antifungal prophylaxis recipients<br>• antifungal prophylaxis drug cost<br>- Fluconazole: $57<br>- Liposomal amphotericin-B: $7,482 and $17,950 based on twice weekly doses of 1 and 3 mg/kg, respectively |
| **Other Infection** | | | |
| **V-HC (cf no V-HC)** | | | |
| McGuirk, 2021 | 1 year | • LOS<br>- ↑ V-HC (72.2 compared to 46.3 days)<br>• readmission per year<br>- ↑ V-HC (3.4 compared to 1.8) | • total cost<br>- ↑ V-HC ($628,765 compared to $339,726) |
| Gilis, 2014 | 5 years | BKV-HC<br>• hospitalization days: | BKV-HC<br>• total cost |

| | | | |
|---|---|---|---|
| | | - 64 days<br>• cidofovir therapy<br>- % receiving cidofovir: 91%<br>- Duration of therapy: 26 days (average 4 doses)<br>- Cumulative dose of cidofovir: 1200 mg | - $181,447 |
| **ARTI (cf no ARTI)** | | | |
| Wattanakamolkul, 2022 | post-HSCT hospitalization: HSCT (index month) to discharge month | • LOS<br>- ↑ ARTI (3.41 compared to 2.38 months for cord; 2.84 compared to 2.28 months for allo-HSCT) | • hospitalization cost<br>- ↑ ARTI ($109,248 compared to $79,520 for cord; $77,907 compared to $72,758 for allo-HSCT) |

Note: (a) control group for one study was GVHD. Abbreviations: aGVHD = acute graft versus host disease; ARTI = acute respiratory tract infection; BKV-HC = Human polyomavirus type 1 (BK) virus associated hemorrhagic cystitis; CADTH = Canadian Agency for Drugs and Technologies in Health; cf = compared; cGVHD = chronic graft versus host disease; CMVd = Cytomegalovirus disease; CMV = Cytomegalovirus; CMVi = Cytomegalovirus infection; ECP = Extracorporeal Photopheresis; ED = Emergency Department; GVHD = graft versus host disease; HSCT = hematopoietic stem cell transplant; ICU = intensive care unit; IST = immunosuppressive medications; LOS = length of stay; NICE = National Institute for Health and Care Excellence, United Kingdom; NS = not specified; TCD = t-cell depletion; Pre-ET = pre-emptive therapy; SR/HR = steroid-refractory or high-risk; SOC = standard of care; V-HC = virus associated hemorrhagic cystitis
